# Association between nutritional level, menstrual-related symptoms, and mental health in female medical students

**DOI:** 10.1371/journal.pone.0235909

**Published:** 2020-07-13

**Authors:** Keiko Fukushima, Noritoshi Fukushima, Hiroki Sato, Jinko Yokota, Keiko Uchida

**Affiliations:** 1 Health Care Center, Tokyo Women's Medical University, Tokyo, Japan; 2 Department of Cardiology, Tokyo Women’s Medical University, Tokyo, Japan; 3 Department of Preventive Medicine and Public Health, Tokyo Medical University, Tokyo, Japan; 4 Department of Cardiology and Clinical Examination, Faculty of Medicine, Oita University, Yufu, Japan; Chiba Daigaku, JAPAN

## Abstract

**Objective:**

Research suggests that medical students as well as women are at greater risk of depression compared to the general population and men. This suggests that female medical students are crucial targets requiring specific monitoring for mental health disorder prevention and intervention. However, little is known regarding the risk factors for poor mental health among this population. Therefore, we investigated whether menstrual symptoms and nutritional status are associated with psychological distress in Japanese female medical students.

**Methods:**

This cross-sectional study assessed 326 female medical students who attended a school medical check-up, which included blood sampling in 2018. The levels of psychological distress were evaluated using the Japanese General Health Questionnaire (J-GHQ)-30. We defined high GHQ scores as GHQ-30 ≥7. We checked dysmenorrhea levels and assessed menstrual symptoms according to the presence of premenstrual syndrome (PMS). Dysmenorrhea was evaluated according to quartiles of the sum of the Menstrual Distress Questionnaire (MDQ). PMS was assessed using the Premenstrual Symptoms Questionnaire (PSQ). We evaluated levels of serum albumin, hemoglobin, ferritin, and lipid metabolite as nutritional factors. A multivariate logistic regression analysis was used to identify the association between menstrual-related symptoms or nutritional factors and the levels of psychological distress.

**Results:**

A total of 45 female medical students (15%) experienced psychological distress. Serum albumin levels were associated with psychological distress, while lipid metabolite levels were not. The intensity of dysmenorrhea and the presence of PMS were associated with psychological distress, independent of nutritional status.

**Conclusion:**

Both menstrual symptoms and nutrition markers were associated with the levels of psychological distress in Japanese female medical students. School doctors and nurses can help improve the mental health of young female medical students by encouraging a healthy diet and checking for the presence of menstrual symptoms.

## Introduction

Many medical students experience depression and suicidal ideation [1] because they are under a high level of stress associated with the immense volume of information to learn, time constraints, examinations, competition, and challenging curricular factors [2–4]. A recent systematic review has reported that the prevalence of depression or depressive symptoms among medical students was 27.2% compared to 9.5% in the general population of 19- to 25-year-olds [1]. Furthermore, there is a significant gender difference in the prevalence of these problematic depressive conditions. Depression and depressive symptoms are twice as more prevalent in women than in men [5–7]. These findings suggest that it is important to identify the factors related to depression and depressive symptoms in female medical students.

Several reasons have been suggested for the increased prevalence of depression in young female medical students; one is menstrual symptoms, and the other is nutritional status. Menstrual-related symptoms, including premenstrual syndrome and menstrual pain have been reported to be associated with mental disorders and depression, particularly in young women [8–11]. Furthermore, lower serum levels of albumin and high-density lipoprotein cholesterol (HDL-C) have been observed in patients with depression compared to healthy controls [12]. Reports also suggest that anemia and lower hemoglobin (Hb) levels are associated with depression [13, 14]. However, the association between nutritional status and depression is still unknown due to inconsistent results [12, 15, 16]. Additionally, although gender differences in the rate of major depression and depressive symptoms peak in adolescence [7], these aforementioned studies regarding nutritional status have focused on middle-aged and elderly participants. To date, little is known about the effects of menstrual symptoms and nutritional status on the mental health of young female medical students.

Therefore, this study aimed to examine the association between menstrual symptoms, nutritional parameters, and psychological distress in Japanese young female medical students.

## Materials and methods

### Study population and design

The participants in this cross-sectional study were students of Tokyo Women’s Medical University. There were 673 medical students who attended a medical check-up at the university between April and July in 2018. At the medical check-up, blood samples were collected from the first-, third-, and sixth-grade students, according to the prescribed university protocol. The exclusion criteria were as follows: (i) presence of chronic diseases such as collagen disease, ulcerous colitis, as well as others, and (ii) insufficient questionnaire data. This study was approved by the Medical Ethics Committee of the School of Medicine, Tokyo Women’s Medical University, Tokyo, Japan (approval no. 5002), and all participants provided written informed consent. The study protocol conforms to the ethical guidelines of the 1975 Declaration of Helsinki and its later amendments.

### Data and sample collection

All data and samples were collected during the medical check-up. Data on demographics and lifestyle factors, including medical history and participation in sporting activities, were collected through a self-administered questionnaire. Height and weight were measured with the participant wearing light clothing. Body mass index (BMI) was calculated as weight (in kilograms) divided by the square of height (in metres). Blood samples were collected in the morning (after the students had fasted overnight). Plasma albumin levels were measured using the bromocresol purple method. The serum levels of triglycerides (TG) and high-density lipoprotein cholesterol (HDL-C) were measured using enzymatic methods. Low-density lipoprotein cholesterol (LDL-C) level was measured using the direct methods.

### The levels of psychological distress

The levels of psychological distress were assessed using the Japanese version of the General Health Questionnaire (J-GHQ-30) [17, 18]. The J-GHQ-30 comprises 30 items, and each item has four subsequently dichotomized response categories, providing a total score of between 0 and 30. The participants were characterized according to their mental state within the last few weeks, including depressive mood, sleeping problems, anxiety, social functioning, well-being, and coping abilities. A high GHQ score was defined as ≥7 of the entire score on J-GHQ-30 [17, 18].

### Menstrual-related symptoms

The premenstrual phase was defined as the 10 days before the first day of menstrual bleeding, while the menstrual phase was defined as the period from the first to the last day of menstruation. Premenstrual symptoms were measured using the Premenstrual Symptoms Questionnaire [19, 20]. This questionnaire was developed and translated into Japanese by Takeda et al. and consists of DSM-IV criteria assessed on a rating scale with degrees of severity. The Premenstrual Symptoms Questionnaire is identical to the Premenstrual Symptoms Screening Tool. The questionnaire asks participants to rate the severity of premenstrual symptoms as ‘not at all’, ‘mild’, ‘moderate’, or ‘severe’. We defined moderate or severe premenstrual symptoms as premenstrual syndrome (PMS). In addition, menstrual symptoms were appraised by the Japanese version of the Menstrual Distress Questionnaire (MDQ) [21]. In this questionnaire, menstrual symptoms were assessed by assigning a score of 1 (no symptoms), 2 (minimal), 3 (mild), 4 (moderate), 5 (strong), or 6 (severe) to each of the 35 items across six of the eight categories (pain, concentration, behaviour change, autonomic reaction, water retention, and negative effect). These scores were summed, and we used the total score (MDQ-score) as the assessment of menstrual symptoms. A higher MDQ-score indicated that the participant was suffering from menstrual symptoms.

### Statistical analysis

Using the Q-Q plot, we checked whether the continuous variables were normally distributed or not. Continuous variables were presented as mean (standard deviation [SD]), or as median (25^th^ percentile, 75^th^ percentile), and categorical variables were presented as frequency and percentage. Continuous variables were compared between the participants with low and high GHQ scores using a student t-test or Mann-Whitney U test. Categorical variables were compared using a Chi-square test. Logistic regression was used to perform univariable and multivariable analyses for the factors influencing psychological distress. The dependent variables were dichotomized as the high or low of the entire J-GHQ-30 score at ≥7 or less; a crude analysis has been presented in model 1. For multiple logistic regression analyses, we made adjustments considering participation in sporting activities as a physical activity factor, age, and school grades as sociodemographic factors and Hb, ferritin, albumin, LDL-C, HDL-C, and TG levels as the nutritional factors in model 2. Model 3 included all model 2 variables plus the presence of PMS and MDQ-scores for menstrual-related symptoms. For analysis, the MDQ-scores were divided into four groups using the 25^th^, 50^th^, and 75^th^ percentiles as cut-off points. All statistical analyses were performed using SPSS version 19.0 (IBM Corp., Armonk, NY, USA). The post-hoc power of the study was estimated using G*Power software (version 3.1) with an α-error of 0.05. The significance level was set at *p* <0.05, and all statistical tests were two-tailed.

## Results

### Characteristics of the study population

**Fig 1:**
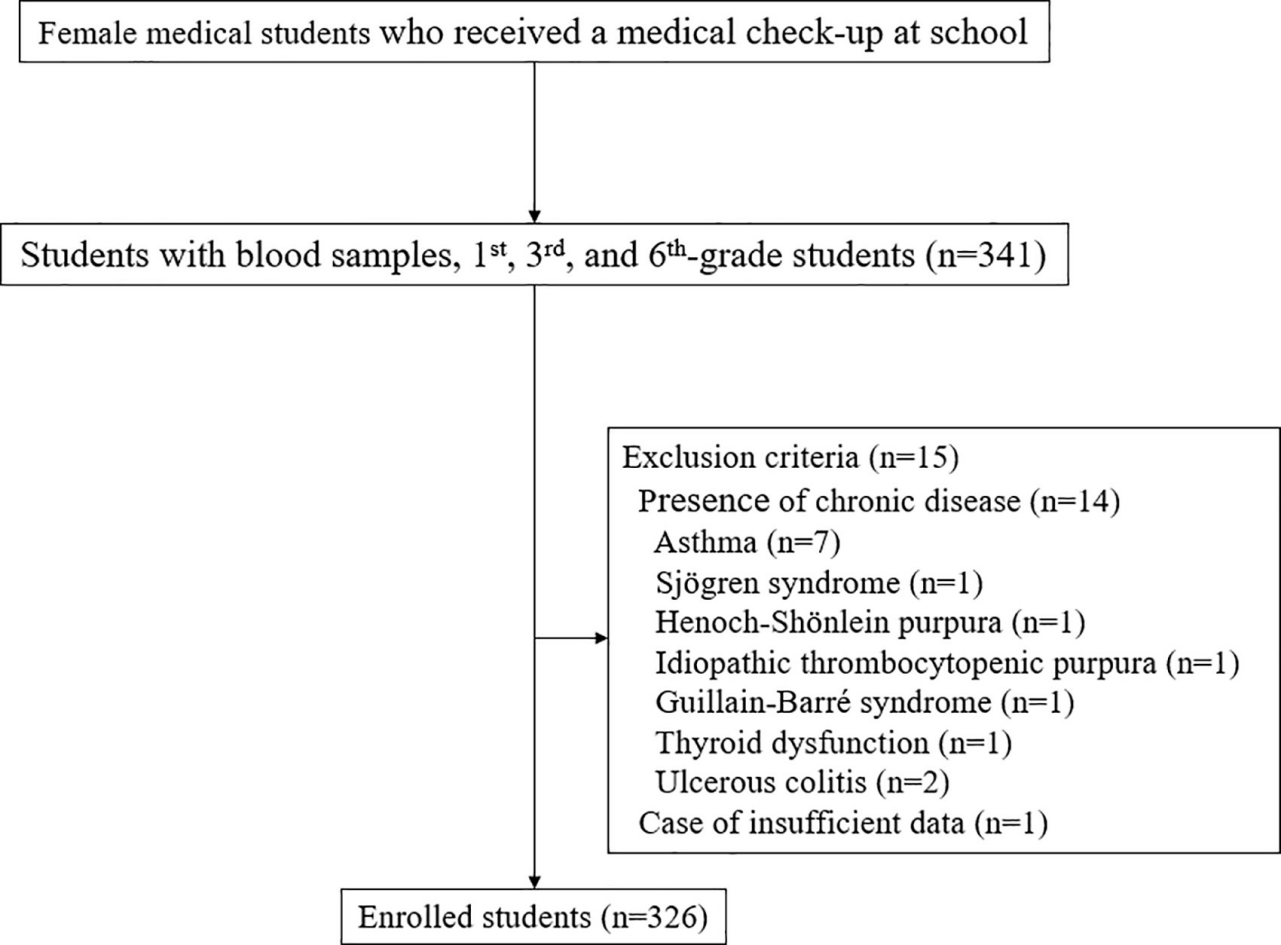
Study flow chart.

In total, 326 Japanese female medical students were included in this study (Fig 1). The characteristics of the study population are summarized in Table 1. Red blood cell count, Hb, hematocrit, and serum albumin levels in the high GHQ group were significantly lower than those in the low GHQ group. There was no significant difference between the low and high GHQ groups with respect to BMI, ferritin, and lipid metabolites (TG, HDL-C, and LDL-C). For menstrual-related symptoms, presence of PMS and the total MDQ score were significantly higher in the high GHQ group compared to the low GHQ group.

**Table 1:** R1. Participant characteristics.

GHQ score	Low-GHQ (n = 277, 85%)	High-GHQ (n = 49, 15%)	P-value
Age, (years)^a^	21.5 (2.7)	22.4 (3.6)	0.057
School grades ^b^			0.822
First year	86.6% (97/112)	13.4% (15/112)	
Third year	83.8% (93/111)	15.3% (17/111)	
Sixth year	82.9% (87/105)	16.2% (17/105)	
Height, (cm)^a^	40.8 (2.5)	39.7 (2.4)	0.293
Weight, (kg)^a^	53.1 (7.0)	53.6 (6.5)	0.627
BMI, (kg/m^2^)^a^	20.9 (2.4)	20.9 (2.3)	0.997
BPs, (mmHg)^a^	108.6 (10.9)	107.1 (11.3)	0.389
BPd, (mmHg)^a^	64.2 (9.3)	64.2 (8.7)	0.863
Participation in sporting activities, Yes^b^	72.7% (200/277)	63.3% (31/49)	0.233
WBC, (×10^3^μL)^a^	6.7 (1.5)	6.6 (1.5)	0.758
RBC, (×10^6^ μL)^a^	4.5 (0.3)	4.4 (0.3)	0.03
Hb, (g/dL)) ^a^	13.5 (0.9)	13.2 (0.9)	0.018
Ht, (%)^a^	40.8 (2.5)	39.7 (2.4)	0.005
Plt, (×10^4^ μL)^a^	27.5 (5.4)	27.0 (5.1)	0.522
Alb, (g/dL)^a^	4.9 (2.7)	4.7 (0.3)	<0.001
AST, (U/L)^c^	17 (15, 20)	17 (15, 19)	0.816
ALT, (U/L)^c^	12 (10, 15)	12 (10, 15)	0.972
Ferritin, (ng/mL)^a^	56.3 (42.8)	56.5 (38.2)	0.975
TG, (mg/dL)^a^	77.6 (39.8)	87.4 (36.5)	0.11
LDL-C, (mg/dL)^a^	102. 2 (31.5)	98.0 (24.7)	0.375
HDL-C, (mg/dL)^a^	74.6 (13.6)	73.4 (13.7)	0.553
Score of GHQ, (score)^c^	0 (0, 2)	9 (8, 12)	<0.001
Premenstrual syndrome, yes^b^	5.1% (14/277)	29.5% (12/49)	<0.001
MDQ score			
Sum, (score)^c^	48 (39, 61)	64 (55, 84)	<0.001
Quartile of sum^b^			<0.001
First quartile	30.3% (84/277)	8.2% (84/49)	
Second quartile	25.6% (71/277)	12.2% (6/49)	
Third quartile	23.1% (64/277)	34.7% (17/49)	
Fourth quartile,	20.9% (58/277)	44.9% (22/49)	

GHQ, General Health Questionnaire; BMI, body mass index; BPs, blood pressure systolic; BPd, blood pressure diastolic; WBC, white blood cell; RBC, red blood cell; Hb, hemoglobin; Ht, hemocrit; Plt, platelet; Alb, albumin, AST, aspartate aminotransferase; ALT, alanine aminotransferase; TG, triglycerides; LDL-C, low density lipid cholesterol; HDL-C, high density lipid cholesterol; MDQ, Menstrual Distress Questionnaire

^a^ Reported as mean (standard deviation)

^b^ Reported as % (numbers)

^c^ Reported as median (25^th^ percentile, 75^th^ percentile)

### Factors related to the high levels of psychological distress

The crude analysis revealed that lower levels of Hb and albumin, presence of PMS, and higher MDS-scores were associated with high levels of psychological distress. After making adjustments considering participation in sporting activities as a physical activity factor, age, and school grades as sociodemographic factors (model 2), a lower level of albumin was significantly associated with high levels of psychological distress, whereas Hb, ferritin, LDL-C, HDL-C, and TG were not. In model 3, which included the menstrual symptoms in addition to the factors considered in model 2, the presence of PMS and higher total MDQ-scores were associated with high levels of psychological distress (Table 2). In addition, lower levels of albumin were independently associated with high levels of psychological distress (Table 2).

**Table 2:** Factors related to psychological distress in female medical students.

	model 1			model 2			model 3		
	OR	95%CI	p-value	OR	95%CI	p-value	OR	95%CI	p-value
Age	1.094	0.996–1.203	0.062	1.068	0.935–1.220	0.331	1.012	0.871–1.176	0.876
School grade									
First year	ref	ref	ref	ref	ref	ref	ref	ref	Ref
Third year	1.182	0.558–2.503	0.662	0.799	0.333–1.913	0.614	0.828	0.317–2.164	0.700
Sixth year	1.264	0.596–2.681	0.542	0.527	0.188–1.479	0.223	0.529	0.171–1.64	0.270
BMI	1.000	0.879–1.139	0.997	0.934	0.806–1.083	0.366	0.961	0.824–1.121	0.613
Participation in sporting activities	0.663	0.350–1.254	0.207	0.534	0.260–1.095	0.087	0.552	0.259–1.178	0.124
Hemoglobin	0.711	0.531–0.950	0.021	0.747	0.526–1.061	0.103	0.686	0.470–1.003	0.052
Ferritin	1.000	0.993–1.007	0.976	1.004	0.996–1.012	0.343	1.005	0.996–1.014	0.283
Albumin	0.444	0.316–0.624	<0.001	0.452	0.308–0.663	<0.001	0.461	0.308–0.692	<0.001
LDL-C	0.995	0.984–1.006	0.372	1.000	0.987–1.012	0.945	1.000	0.987–1.014	0.960
HDL-C	0.993	0.971–1.016	0.551	1.000	0.975–1.026	0.992	1.003	0.976–1.030	0.845
TG	1.006	0.999–1.013	0.114	1.007	0.998–1.015	0.118	1.009	1.000–1.019	0.058
PMS	6.093	2.619–14.174	<0.001				3.390	1.086–10.586	0.036
MDQ score									
First quartile	ref	ref	ref				ref	ref	Ref
Second quartile	2.242	0.559–9.000	0.255				2.697	0.631–11.525	0.181
Third quartile	5.873	1.626–21.215	0.007				6.409	1.646–24.957	0.007
Fourth quartile	9.397	2.702–32.679	<0.001				7.377	1.805–30.144	0.005

Model 1, unadjusted; model 2 is adjusted for BMI, participation in sporting activities as a physical activity factor, age, and school grades as sociodemographic factors, and Hb, ferritin, albumin, LDL-C, HDL-C, and TG as nutritional factors; model 3 includes all model 2 variables plus the presence of PMS and MDQ-score as menstrual-related symptoms.

The levels of albumin are standardized. ORs are computed per standard deviation increment.

CI, confidence interval; OR, odds ratio; BMI; body mass index, LDL-C; low density lipoprotein-cholesterol, HDL-C, high density lipoprotein-cholesterol; TG, triglycerides; PMS; premenstrual syndrome; MDQ, Menstrual Distress Questionnaire; TG, triglycerides; Hb, hemoglobin

With regard to the statistical power in model 1, the calculated statistical power of Hb, albumin, the presence of PMS, and MDS-scores were 0.057, 0.999, 0.970, and 0.999, respectively. Moreover, in model 2, the calculated statistical power of Hb and albumin were 0.059, 0.999. Finally, in model 3, those of Hb, albumin, the presence of PMS, and MDS-scores were 0.054, 0.996, 0.850, and 0.996, respectively.

## Discussion

In this study, we examined the influence of menstrual symptoms and nutritional status on high levels of psychological distress in young female medical students in Japan. Our main findings were: (i) 15% of the female medical students experienced psychological distress, (ii) there were associations between menstrual-related symptoms and the levels of psychological distress, (iii) serum albumin levels were significantly associated with the levels of psychological distress, and (iv) these associations remained significant after adjusting for confounding factors.

In the present study, the prevalence of psychological distress among female medical students was 15%. It has been reported that the prevalence of major depressive episodes was 2.0% among Japanese undergraduates, as calculated by the Patient Health Questionnaire-9 (PHQ-9) [22], and 6.6% in the general female Japanese population aged 20–39 years, calculated using the Center for Epidemiological Studies Depression scale (CES-D) [23]. Moreover, the National Health and Nutrition Examination Survey (NHANES) revealed that the prevalence of depression symptoms was 7.4% in the general American population aged 18–39 years, as determined using the PHQ-9 [24]. Although this study did not use the same questionnaire to assess psychological distress, our results may have shown a higher prevalence of depressive symptoms in Japanese female medical students compared to Japanese non-medical students and the general population. However, the prevalence was lower than that in medical students reported by a previous systematic review (27%) [2]. In the general population, the prevalence of mood and anxiety disorders in East-Asian countries is lower than that in Western countries [25, 26]. In Asian medical students, the prevalence of depression varies broadly, being 10.3% in Korea [27], 21.4% in Thailand [28], and 29% in China [29]. There are a few studies on the prevalence of depression in Japanese medical students. Ohtsu et al. reported that poor mental health status, as assessed by GHQ-12, was observed in 48.8% of fourth-grade female medical students in Japan [30]. In the Japanese medical educational curriculum, the fourth-grade involves an important preparation period before clinical training, including computer-based testing and objective structured clinical examinations as common achievement tests; this may have resulted in a higher prevalence of depressive symptoms. In this regard, the participants in this study included sixth-grade students, who were facing challenges in the crucial preparation period before the state examination for a license to practice medicine. Indeed, the prevalence rate of psychological distress in sixth-grade medical students was higher than that in other grades, but this difference was not statistically significant. In this study, the prevalence of psychological distress may be slightly underestimated by including participants from other grades (i.e., first- and third-grade). Future studies from all six academic years using a unified questionnaire are needed to identify the prevalence of depression in Japanese female medical students.

We found that lower albumin levels were negatively associated with depressive symptoms in female medical students. A potential underlying mechanism for this relationship between serum albumin levels and depression could be explained. Lower serum albumin levels contribute to oxidative stress dysregulation and an increased inflammation that are associated with depression and lower antioxidant capacity [31, 32].

Conversely, the crude analysis revealed that lower levels of Hb were associated with high levels of psychological distress. Previously, Chen et al. reported that lower Hb levels were correlated with depressive symptoms, although the mean levels of Hb did not indicate anemia [14]. However, Hb levels tended to be associated with mental health disorders after adjustments for exercise habits, age, academic years, nutrition factors, and menstrual-related factors, but the association was not statistically significant. In this regard, the calculated statistical power of Hb in this study was quite low, while those of albumin, the presence of PMS, and MDS-scores were all over 0.85; this appeared to be substantially sufficient. Although the results of post hoc power analysis need to be interpreted with caution [33–36], the relatively lower prevalence of female distressed medical students in this study may have weakened its statistical power of the study, particularly for the assessment of Hb. Furthermore, reports suggest that the levels of Hb are associated with mental health in elderly and middle-aged participants. In young female medical students, the association between the levels of Hb and mental health may be confounded by age, physical activity, sociodemographic factors, nutritional factors, and menstrual related factors.

We also found that higher levels of menstrual symptoms and the presence of premenstrual syndrome were associated with high levels of psychological distress. These results are consistent with those reported previously [8–11]. To the best of our knowledge, this is the first report on Japanese female medical students, a population at risk of psychological distress. A potential underlying mechanism for menstrual symptoms may be neuroendocrine dysfunction. Fukuda et al. reported that bradykinin was associated with anxiety problems. Additionally, serotonin dysregulation, a factor of major depression, has been suggested as an etiological factor for premenstrual syndrome [11]. These sources of neuroendocrine dysfunction may be related to depressive symptoms.

Furthermore, it is well known that in adolescent women, dysmenorrhea is often caused by endometriosis and polycystic ovary syndrome (PCOS). It has also been reported that women with endometriosis or PCOS have a higher prevalence of depression [37, 38]. Many treatments including nonsteroidal anti-inflammatory drugs and oral contraceptives, are reported to be effective in menstrual-related symptoms, including premenstrual dysphoric disorder and dysmenorrhea [39, 40]. Furthermore, mental health care teams and wellness advisors in medical college are important as mental health services for medical students [41]. Given that female students with premenstrual symptoms caused by endometriosis and PCOS are often screened earlier or provided with a referral to gynecology from a mental health care team or wellness advisors in school, treatment of such gynecological diseases may contribute to improvements in mental health among female students.

This study has several limitations. First, it was a single university study with a relatively small sample size; this may limit the generalizability of the findings. Second, this was a cross-sectional study, and thus, we could not infer associations between the nutritional status and menstrual-related symptoms with high levels of psychological distress; further longitudinal studies are necessary to clarify this aspect. Finally, we did not analyze data pertaining to physical activity, which is considered to be associated with mental health [42]. However, we did adjust for participation in sporting activities (more than 2 hours and 2 times in a week) as physical activity.

## Conclusions

The levels of psychological distress in female medical students may be associated with nutritional markers such as albumin, and with menstrual-related symptoms. It is important to improve dietary habits and control menstrual-related symptoms in female medical students to improve mental health. School doctors and nurses can play a role in ameliorating mental health in young female medical students by encouraging a healthy diet and checking for the presence of menstrual symptoms.
