# Correction: Association between nutritional level, menstrual-related symptoms, and mental health in female medical students

**DOI:** 10.1371/journal.pone.0317846

**Published:** 2025-01-15

**Authors:** Keiko Fukushima, Noritoshi Fukushima, Hiroki Sato, Jinko Yokota, Keiko Uchida

In the Menstrual-related symptoms subsection of Materials and Methods, the references cited in the second up to the seventh sentences are incorrect. The sentences should have been written as “…Premenstrual symptoms were measured using the Premenstrual Symptoms Questionnaire [19, 20]. This questionnaire was developed by Miyaoka et al, which was based on DSM-IV criteria and was modified from the premenstrual symptoms screening tool developed by Steiner et al [21]. The questionnaire asks participants to rate the severity of premenstrual symptoms as ‘not at all’, ‘mild’, ‘moderate’, or ‘severe’. We defined moderate or severe premenstrual symptoms as premenstrual syndrome (PMS). In addition, menstrual symptoms were appraised by the Japanese version of the Menstrual Distress Questionnaire (MDQ) [22]…”.

As a result of the correction to the Menstrual-related symptoms, References 19–22 should have been as follows:

19. Miyaoka Y, Akimoto Y, Ueda K, Ujiie Y, Kametani M, Uchiide Y, et al BioPsychoSocial Medicine 2011; 5:5 10.1186/1751-0759-5-5

20. Miyaoka Y, Akimoto Y, Kamo T, Ueda K: The reliability and validity of the newly developed PMDD scale. J Jp Soc Psychosom Obstet Gynecol 2009; 14:194–201, (In Japanese). doi: https://doi.org/10.18977/jspog.14.2_194

21. Steiner M, Macdougall M, Brown ET: The premenstrual symptoms screening tool (PSST) for clinicians. Arch Womens Ment Health 2003;6:203–209. doi:10.1007/s00737-003-0018-4

22. Goto Y, and Okuda H. Perimenstrual symptoms and it’s management—Assessment with Menstrual Distress Questionnaire -. Bull Fac Health Sci, Okayama Univ Med Sch 16: 21–30, 200. http://doi.org/10.18926/15180
